# ASO Author Reflections: Standardized and Reproducible Surgical Steps of Nerve-Sparing Radical Hysterectomy

**DOI:** 10.1245/s10434-026-19511-6

**Published:** 2026-03-23

**Authors:** Yusuke Tanaka

**Affiliations:** Department of Obstetrics and Gynecology, Faculty of Medicine, Oita University, Yufu, Oita Japan

## Past

In the SHAPE trial,^1^ simple hysterectomy for patients with low-risk early-stage cervical cancer was not inferior to radical hysterectomy with respect to the survival rate. Based on the result, simple hysterectomy is recommended for patients with low-risk early-stage uterine cervical cancer, which can now be considered a new standard of care. However, for patients with cervical cancer > 2 cm with “non-SHAPE” criteria, radical hysterectomy is still recommended as the standard primary treatment.

## Present

We demonstrated both the anatomical highlights and a step-by-step procedure for nerve-sparing radical hysterectomy.^2^ In this report, we emphasized the importance of the precise development of the avascular space because it is foundational to the standardization of radical hysterectomy. However, in practice, the surgical field and dissection line during radical hysterectomy differ among surgeons.^2–4^ This is because the methodology for the development of avascular spaces differs. In radical hysterectomy, precise identification and development of the avascular spaces represent one of the most technically demanding and essential components of the procedure. However, in cadaveric models—particularly in recent reports describing surgical techniques based solely on cadaveric demonstrations^3,4^—the absence of active bleeding may mask incomplete identification of the spaces, potentially creating a misleading impression of technical adequacy. Some authors have raised concerns that variations in the creation of avascular spaces may exist among different surgeons, and the reliance on uncertain anatomical structures for defining excision borders appears to lack precision.^5^ Avascular spaces cannot be identified without adequate surgical traction. In the absence of appropriate surgical traction, surgical instruments reach the weakest part, which can result in the creation of false tissue planes.

## Future

Nerve-sparing radical hysterectomy requires deep knowledge of pelvic neurovascular anatomy. Understanding the anatomical relationship between the deep uterine vein, hypogastric nerve, inferior hypogastric plexus, and bladder branch is essential. Lack of proper anatomical knowledge and adequate surgical skills are associated with not only the risk of hypogastric nerve injury but also unnecessary bleeding during surgery, which may lead to unintentionally less radical surgery. Surgeons should be trained to precisely develop the retroperitoneal avascular spaces.
